# DNA-based stable isotope probing coupled with cultivation methods implicates *Methylophaga* in hydrocarbon degradation

**DOI:** 10.3389/fmicb.2014.00076

**Published:** 2014-02-27

**Authors:** Sara Mishamandani, Tony Gutierrez, Michael D. Aitken

**Affiliations:** ^1^Department of Environmental Sciences and Engineering, Gillings School of Global Public Health, University of North CarolinaChapel Hill, NC, USA; ^2^Centre for Marine Biodiversity and Biotechnology, School of Life Sciences, Heriot-Watt UniversityEdinburgh, UK

**Keywords:** hydrocarbon degradation, stable isotope probing, *Methylophaga*, *Alcanivorax*, *n*-hexadecane, marine environment

## Abstract

Marine hydrocarbon-degrading bacteria perform a fundamental role in the oxidation and ultimate removal of crude oil and its petrochemical derivatives in coastal and open ocean environments. Those with an almost exclusive ability to utilize hydrocarbons as a sole carbon and energy source have been found confined to just a few genera. Here we used stable isotope probing (SIP), a valuable tool to link the phylogeny and function of targeted microbial groups, to investigate hydrocarbon-degrading bacteria in coastal North Carolina sea water (Beaufort Inlet, USA) with uniformly labeled [^13^C]*n*-hexadecane. The dominant sequences in clone libraries constructed from ^13^C-enriched bacterial DNA (from *n*-hexadecane enrichments) were identified to belong to the genus *Alcanivorax*, with ≤98% sequence identity to the closest type strain—thus representing a putative novel phylogenetic taxon within this genus. Unexpectedly, we also identified ^13^C-enriched sequences in heavy DNA fractions that were affiliated to the genus *Methylophaga*. This is a contentious group since, though some of its members have been proposed to degrade hydrocarbons, substantive evidence has not previously confirmed this. We used quantitative PCR primers targeting the 16S rRNA gene of the SIP-identified *Alcanivorax* and *Methylophaga* to determine their abundance in incubations amended with unlabeled *n*-hexadecane. Both showed substantial increases in gene copy number during the experiments. Subsequently, we isolated a strain representing the SIP-identified *Methylophaga* sequences (99.9% 16S rRNA gene sequence identity) and used it to show, for the first time, direct evidence of hydrocarbon degradation by a cultured *Methylophaga* sp. This study demonstrates the value of coupling SIP with cultivation methods to identify and expand on the known diversity of hydrocarbon-degrading bacteria in the marine environment.

## Introduction

Hydrocarbon-degrading bacteria comprise an important component to the total microbial diversity in the marine environment, contributing significantly to the degradation and ultimate removal of hydrocarbons from the marine water column and sediment. Considering the enormous volumes of hydrocarbons that enter the oceans each year through natural seepage, anthropogenic activities and other sources, the fact that the sea surface is not covered in a layer of oil is largely due to the presence and activities of these types of bacteria. Species of hydrocarbon-degrading bacteria, belonging to over 20 genera and distributed across some of the major bacterial Classes (*Alpha*-, *Beta*- and *Gammaproteobacteria*; *Actinomycetes*; *Flexibacter-Cytophaga-Bacteroides*), have been isolated and described (Floodgate, 1995; Head and Swannell, 1999; Head et al., 2006; Yakimov et al., 2007). To our knowledge, the marine environment is the only place where we find bacteria with the ability to utilize hydrocarbons almost exclusively as a sole source of carbon and energy. So-called “hydrocarbon specialists”, or “obligate oil-degraders”, these organisms are ubiquitous in the ocean, often found at <0.1% abundance of the total microbial community in seawater, and becoming strongly selected for and increasing to levels constituting up to 90% of the total microbial community upon exposure to crude oil or its refined petrochemical products (Röling et al., 2002, 2004; Teira et al., 2007). This hydrocarbon-elicited bloom in microbial-expressed hydrocarbon catabolizing potential is one of the ocean's inherent mechanisms to keeping the total load of hydrocarbons to background levels, in-turn helping to mitigate the potential detrimental effects that these chemical pollutants can pose to marine life.

Whilst our knowledge on the diversity of hydrocarbon-degrading bacteria in the ocean has progressed, it is far from complete, and novel taxa continue to be discovered. One bacterial genus that has proven contentious with respect to whether any of its members might be capable of degrading hydrocarbons is *Methylophaga*. Members of this genus are strictly aerobic and moderately halophilic, belonging to the *Piscirickettsiaceae* family in the *Gammaproteobacteria*, and exhibit an exclusive requirement for C_1_ sources (methanol, methylamine, dimethylsulfide) as sole growth substrates, with the exception of some species that are also capable of utilizing fructose (Janvier and Grimont, 1995). A few studies have reported the enrichment of *Methylophaga* spp. in oil-contaminated field samples and in laboratory experiments with oil (Röling et al., 2002; Yakimov et al., 2005; Coulon et al., 2007), but whether those organisms could degrade hydrocarbons was not addressed in those studies. Recently, Vila et al. (2010) isolated a *Methylophaga* species, designated strain AF3, from a beach impacted by the *Prestige* oil spill and showed it to grow in a synthetic seawater medium amended with high molecular weight polycyclic aromatic hydrocarbons (PAHs). However, since the medium was also amended with nutritionally-rich Luria-Bertani medium, the assumption that strain AF3 could grow on PAHs was unsubstantiated. Hence, compelling evidence showing a *Methylophaga* species to degrade hydrocarbons remains lacking.

One method that circumvents the requirement to isolate microorganisms in order to assess their metabolic and physiological characteristics is stable isotope probing (SIP). This method has been used successfully on environmental samples to identify a target microbial group(s) based on their ability to perform a specific metabolic process, thereby being able to link the phylogenetic identity of an organism to its function (Dumont and Murrell, 2005). An added advantage of this technique is its ability to identify target members of a microbial community that are not amenable to cultivation in the laboratory. In this study, we investigated hydrocarbon-degrading bacteria in surface seawater on the North Carolina coast using DNA-based SIP with uniformly labeled [^13^C]*n*-hexadecane and identified *Alcanivorax* and several other taxa, including *Methylophaga*, in the isolated ^13^C-labeled “heavy” DNA. We subsequently isolated a strain representing this SIP-identified *Methylophaga*, designated strain SM14, which was found capable of growing on *n*-hexadecane as a sole source of carbon and energy. This work, which couples DNA-SIP with cultivation-based methods, for the first time reveals direct evidence implicating a *Methylophaga* species with the ability to utilize a hydrocarbon as a growth substrate.

## Materials and methods

### Field sample

During a field trip 1 mile offshore from the Beaufort Inlet (34° 33.42′ N, 76° 51.06′ W), North Carolina, USA on 27 August 2010, ca. 20 L of surface seawater was collected into pre-autoclaved polypropylene (Nalgene) bottles and stored at 4°C. On the following day, ca. 18 L of the water sample was filtered through 0.2-μm Nucleopore filters and the retained biomass resuspended in ONR7a medium (Dyksterhouse et al., 1995) to a total volume of ca. 100 ml to act as the inoculum for use in enrichment, mineralization, degradation and SIP experiments (described below).

### SIP incubations

SIP incubations were performed as described previously (Gutierrez et al., 2013). Briefly, 16 125-ml autoclaved glass screw-top Erlenmeyer flasks with caps lined with aluminum foil to prevent the adsorption of hydrocarbons were prepared. Each flask contained 15 ml of ONR7a medium, 1mg of labeled (^14^C or ^13^C) and/or unlabeled *n*-hexadecane, and 5 ml of inoculum. [U-^13^C] *n*-hexadecane was obtained from Sigma-Aldrich (United States). For SIP, duplicate flasks were prepared with 1 mg of [U-^13^C] *n-hexadecane*, and a second set of duplicates was prepared with 1mg of the unlabeled counterpart. To determine the endpoint of each SIP experiment, the mineralization of [U-^14^C] *n*-hexadecane was measured in triplicate flasks by liquid scintillation counting of ^14^CO_2_ trapped in KOH-soaked filter paper over time, as described below. An additional set of triplicate flasks was used to monitor the disappearance of unlabeled *n*-hexadecane by gas chromatography–mass spectrometry (GC–MS). Samples were taken periodically from these flasks for DNA extraction and subsequent measurement of the abundance of target organisms (by qPCR as described below) identified through SIP. Triplicate flasks of acid-killed controls (pH ≤ 2) containing unlabeled *n*-hexadecane were prepared by adding 85% phosphoric acid (ca. 0.7 ml per flask). All flasks were incubated on an orbital shaker (250 rpm; 21°C) in the dark. At the endpoint of each SIP incubation—defined as the time when the extent of mineralization of the ^14^C-labeled *n*-hexadecane began to approach an asymptote—whole DNA from the total volume in the paired flasks amended with the [U-^13^C] *n*-hexadecane and the corresponding paired set with unlabeled *n*-hexadecane was extracted using the method of Tillet and Neilan (2000).

To monitor the mineralization of [U-^14^C] *n*-hexadecane, each triplicate flask contained the ^14^C-labeled compound to 20,000 dpm and 2.5 mg of unlabeled *n*-hexadecane. Killed controls (in triplicate) were also prepared by adding 85% phosphoric acid to pH ≤ 2 prior to inoculation. For the CO_2_ trap, a sterile glass test tube (12 × 75 mm) containing a piece of filter paper saturated with 60 μl of 2 M KOH was inserted into each flask. The filter paper from each flask was removed daily and the captured ^14^C from any ^14^CO_2_ respired was counted on a Packard (Meriden, CT, USA) Tri-Carb liquid scintillation analyser (model 1900TR). The KOH-saturated filter paper from each flask was replaced at each sampling point for the course of the experiment. The percentage of ^14^C mineralized for [U-^14^C] *n*-hexadecane was calculated by subtracting the triplicate values for the acidified controls from those of the experimental and then dividing by the total dpm of ^14^C added.

### CsCL gradient ultracentrifugation and identification of ^13^C-enriched DNA

To separate ^13^C-enriched and unenriched DNA, total extracted DNA from each sample was added to cesium chloride (CsCl) solutions (1.72 g ml^−1^) for isopycnic ultracentrifugation and gradient fractionation, as previously described (Jones et al., 2011). As an internal standard for unlabeled DNA, 5 μl of purified *Escherichia coli* DNA (ca. 40 ng ml^−1^) was added and mixed into each tube prior to ultracentrifugation. Each fraction was then analyzed by denaturing gradient gel electrophoresis (DGGE) to visualize the separation of DNA. For this, PCR amplification of each fraction was carried out with primers 63f-GC (Marchesi et al., 1998) and 517r (Muyzer et al., 1993) using a PCR program as described by Yu and Morrison (2004). PCR products were confirmed on a 1.5% (w/v) agarose gel alongside a HindIII DNA ladder (Invitrogen, Carlsbad, CA, USA). DGGE was performed using 6.5% acrylamide gels containing a denaturant range of 30–60%. After electrophoresis for 16 h at 60°C and 60 V, gels were stained with ethidium bromide (1:25,000 dilution; 15 min). Gel images were captured and visualized using the GNU Image Manipulation Program (GIMP; version 2.6.8).

### 16S rRNA gene libraries of ^13^C-enriched DNA

16S rRNA clone libraries, each comprising 96 clones, were prepared from combined fractions containing the ^13^C-enriched DNA from each of the duplicate SIP incubations. For this, the general eubacterial primers 27f and 1492r were used for amplification of the 16S rRNA gene and then partially sequenced using primer 27f (Wilmotte et al., 1993) at the Beckman Coulter Genomics sequencing facility (Danvers, MA, USA). The ^13^C-enriched heavy DNA fractions were selected based on the DGGE evidence, as discussed below. After excluding vector sequences, poor-quality reads and chimeras, clone sequences were grouped into operational taxonomic units (OTUs) based on applying a 97% sequence identity cutoff. The complete linkage clustering and dereplicate tools available at the Pyrosequencing Pipeline tool of RDP-II (Cole et al., 2009) were used to select representative sequences for dominant OTUs identified in each of the libraries. Near-complete 16S rRNA gene sequences for the represented sequences were obtained at the University of North Carolina-Chapel Hill Genome Analysis Facility. Sequencher 4.8 (Gene Codes Corp., Ann Arbor, MI, USA) was used to edit and assemble these sequences, and the BLASTn search program and RDP-II (Maidak et al., 1999) were used to check for close relatives and phylogenetic affiliation.

### Real-time quantitative PCR

To quantify sequences in the dominant OTUs, primers for real-time quantitative PCR (qPCR) were developed using the Probe Design and Probe Match tools of ARB, as previously described (Gutierrez et al., 2011). The Probe Check tool of RDP-II was used to confirm primer specificity, and the optimal annealing temperature of each primer pair was determined using an Eppendorf (Hauppauge, NY, USA) or Applied Biosystems (Foster City, CA. USA) Mastercycler gradient thermal cycler. The template for these reactions, and for the construction of respective standard curves for qPCR, was either a plasmid containing a representative sequence that had been linearized using PstI (New England BioLabs, Ipswich, MA, USA), or a PCR amplicon of the 16S rRNA gene, and purified using the QIAquick nucleotide removal kit (Qiagen, Valencia, CA, USA). The primer pairs, their optimal annealing temperature, amplification efficiency (Pfaffl, 2001), detection limit and RDP hits are shown in Table 1. To confirm the fractions from the DGGE profiles that corresponded to unlabeled DNA, *E. coli* primers ECP79f (5′-GAAGCTTGCTTCTTTGCT-3′) and ECR620r (5′-GAGCCCGGGGATTTCACA-3′) were used to quantify the abundance of the *E. coli* 16S rRNA genes in each fraction. An annealing temperature of 55°C was used for the qPCR program employing these primers (Sabat et al., 2000).

**Table 1:** **Quantitative PCR primers developed and used in this study**.

**Target OTU**	**Primer name**	**Primer sequence (5′ → 3′**)	**T_M_ (°C)^a^**	**qPCR standard^b^**	**Amplicon length**	**Amp. Eff.^c^**	**Detect. limit^d^**	**RDP hits^e^**
1	Alc-411	CSKTGGAGTACTTGACGT	58	HEX19	195	1.82	5	21
	Alc-604r	CTGCACTCTAGCYTGCCA						448(6)
4	Met-126f	GGGATCTGCCTGACAGTGGG	60	HEX76	90	1.63	1	88
	Met-214r	GGTTCATCTGTCAGCGTGAG						96(83)

a Empirically determined PCR annealing temperature.

b Representative clone sequences used to generate standard curves. Names are as in Figures 3, 5.

c Amp. Eff., amplification efficiency (Pfaffl, 2001) with OTU-specific primers.

d Detection limit of each qPCR assay expressed as number of 16S rRNA gene copies per milliliter of culture.

e Number of sequences returned by the Ribosomal Database Project II release 10.18 (Cole et al., 2009)(excluding sequences from this study) with no mismatches to primer pairs. Values in parentheses are the total hits that each pair of primers target.

Purified DNA from time-series incubations with unlabeled hydrocarbon was quantified using a NanoDrop ND-3300 fluorospectrometer (Thermo, Waltham, MA, USA) and the Quant-iT Picogreen double-stranded DNA (dsDNA) kit (Invitrogen). As duplicates of the separated ^12^C- and ^13^C-labeled incubations for each of the three SIP incubations displayed similar distributions of DNA in the fractions, as well as similar DGGE profiles, only the replicate incubation whose fractions contained the highest total amount of DNA was used for further analyses. SIP-identified sequences were quantified in each separated SIP fraction using at least duplicate reactions by qPCR, as described previously (Singleton et al., 2006). Single reactions were performed on each triplicate DNA extraction (from triplicate samples) from the time series containing unlabeled hydrocarbon.

### Isolation and degradation experiments

The detection of *Methylophaga* sequences in the heavy DNA clone library from SIP prompted us to isolate these organisms in order to further verify their potential to degrade hydrocarbons. For this, a fresh inoculum was prepared (as described above) from the same batch of North Carolina surface seawater and used to inoculate three 250-ml Erlenmeyer flasks, each containing 50 ml of ONR7a medium amended with methanol supplied via the vapor phase, as previously described (Paje et al., 1997). Methanol, as the sole carbon and energy source, was used in order to preferentially enrich for methylotrophs. After 1-week incubation (250 rpm; 21°C) in the dark, samples (50 μl) from each flask were streaked directly onto ONR7a agar plates and stored in a desiccator containing a small beaker containing ca. 50 ml of methanol. Colonies displaying distinct colonial morphologies were picked and subcultured onto fresh ONR7a agar medium (amended with methanol via the vapor phase) until pure cultures were obtained and stored in glycerol (30% v/v) at −80°C. One isolate, designated strain SM14, was selected for further study.

The potential of strain SM14 to grow on *n*-hexadecane as the sole carbon and energy source was determined in acid-washed (0.1 N HCl) 250-ml autoclaved glass screw-top Schott bottles with caps lined with aluminium foil to prevent the adsorption of hydrocarbons. Each bottle contained 50 ml of ONR7a medium and 1mg of unlabeled *n*-hexadecane (>99% purity). One set of triplicate bottles was inoculated with strain SM14 cells that had been washed several times with sterile ONR7a broth. Uninoculated controls, acid-killed controls and bottles that were inoculated, but without any added *n*-hexadecane, were also prepared. All incubations were conducted in triplicate and incubated in the dark with shaking (150 rpm) at 25°C. Growth was monitored spectrophotometrically by taking triplicate measurements of the culture medium periodically at an optical density of 600 nm. Concentrations of *n*-hexadecane were measured by gas chromatography (GC). For this, triplicate samples (1 ml) were taken at each time point and extracted with 2 ml of ethyl acetate (EA). Heptanone, as internal standard, was added to each of the non-aqueous EA extracts prior to injection (3 μl) into a Hewlett Packard 5890 series II GC equipped with an Equity 1 (Supelco) column (60 m × 0.32 mm i.d.) and a flame ionization detector. The operating conditions were as follows: column temperature initially 60°C for 1 min, then 60–250°C at 18°C/min, followed by holding at 250°C for 18.5 min; injector temperature at 300°C; detector temperature at 310°C; and carrier gas He (1.0 ml min^−1^).

### Phylogenetic tree

The 16S rRNA sequences of the isolated strain and SIP-identified representative sequences were aligned using CLUSTAL_X (Thompson et al., 1994) with close relatives as determined by RDP and BLASTn searches of GenBank. A neighbor-joining tree was constructed with bootstrapped replication (1000 times) and *Escherichia coli* 0157:H7 (AY513502) was used as an outgroup.

### Nucleotide sequence accession numbers

The following accession numbers were submitted to GenBank for ^13^C-enriched DNA from SIP experiment with *n*-hexadecane: *Alcanivorax* sp. clone HEX19 (KF875698) and *Methylophaga* sp. clone HEX76 (KF790924). The accession number for *Methylophaga* sp. strain SM14 is KF790925.

## Results

### Incubations with labeled and unlabeled n-hexadecane

**Figure 1:**
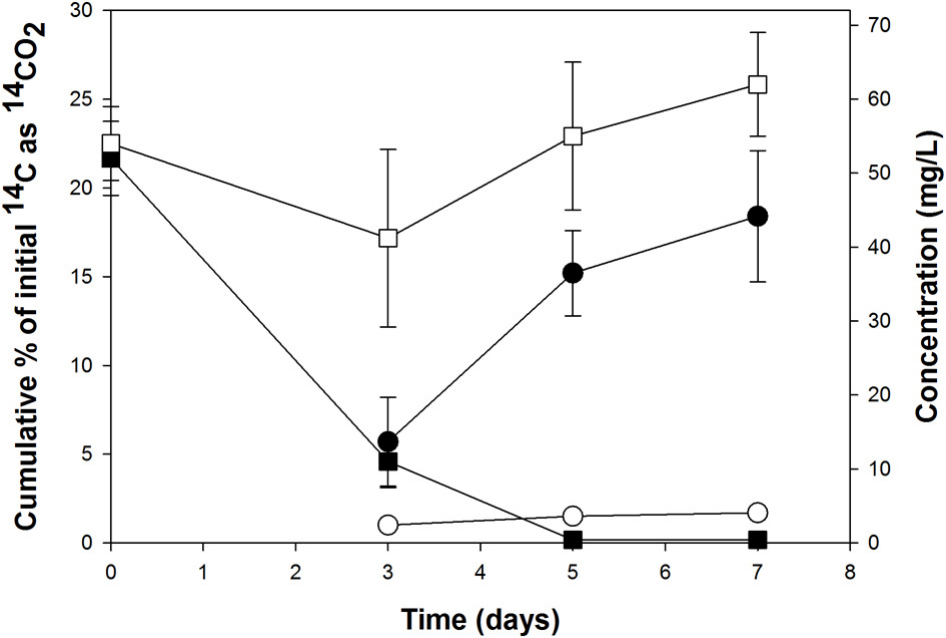
**Cumulative ^14^CO_2_ recovered from incubations with [^14^C]*n*-hexadecane (circles) and disappearance of *n*-hexadecane as measured by GC-MS (squares) by the North Carolina surface seawater field sample.** The endpoint for this SIP incubation was determined to be 7 days. Each data point is the mean ± standard deviation from triplicate incubations. Filled symbols represent live cultures (non-acid treated); open symbols represent acid-inhibited controls. Some error bars are smaller than the symbol.

During the SIP experiment, incubations containing unlabeled or ^14^C-labeled *n*-hexadecane were run in parallel to measure, respectively, for the disappearance and mineralization of this hydrocarbon. As shown in Figure 1, complete removal of *n*-hexadecane occurred by day 5, whereas mineralization of ^14^C associated with *n*-hexadecane continued up until day 7. The endpoint selected for the extraction of DNA from the ^13^C incubations was 7 days, which corresponded to slowing of the mineralization rate for the *n*-hexadecane. The DNA extracts (from each of the duplicate ^13^C incubations) was subjected to isopycnic ultracentrifugation to isolate the ^13^C-enriched “heavy” DNA for subsequent analysis.

### DNA gradient ultracentrifugation and identification of labeled 16S rRNA genes

**Figure 2:**
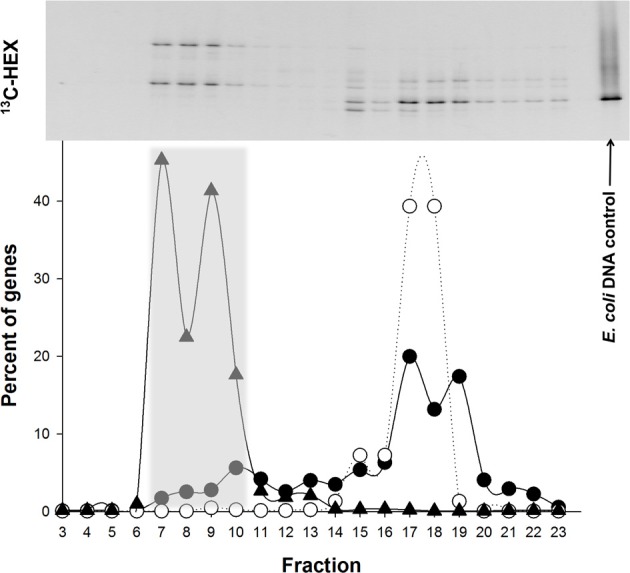
**Distribution of the “heavy” and “light” DNA in separated SIP fractions.** (Top) DGGE image of bacterial PCR products from separated [^13^C]*n*-hexadecane fractions, with decreasing densities from left to right. The position of unlabeled *E. coli* DNA, which was used as an internal control in the isopycnic centrifugation, is shown on the right. (Bottom) Distribution of qPCR-quantified 16S rRNA gene sequences is shown below the DGGE image for *Alcanivorax* (triangles) and *Methylophaga* (solid circles) in fractions from the [^13^C]*n*-hexadecane incubations. The distribution of qPCR-quantified 16S rRNA gene sequences for *E. coli* is also shown (open circles) in fractions from the ^13^C incubation. Gene copies in a fraction are presented as a percentage of the total genes quantified in the displayed range of fractions. Data points are aligned with equivalent fractions of the DGGE image.

DGGE analysis of the fractions from the SIP incubations showed clear evidence of isotopic enrichment of DNA in [^13^C]*n*-hexadecane incubations, separation of ^13^C-labeled and unlabeled DNA, and different banding patterns between the ^13^C-enriched and unenriched DNA fractions (Figure 2). For the ^13^C incubations shown in Figure 2, fractions 7–10 were combined and used to construct the 16S rRNA gene clone library. Fractions from the duplicate gradient (i.e., of the duplicate ^13^C incubation) were combined and similarly manipulated (data not shown). After excluding vector sequences, poor sequence reads, chimeras, and singleton sequences, the clone library constructed from pooled ^13^C-enriched DNA comprised 85 sequences. Table 2 shows the OTU representation in the clone library together with the phylogenetic affiliation based on a BLASTn search in GenBank. Of the 85 sequences, 5 OTUs were identified based on a >97% sequence identity cutoff. OTU-1 (71 sequences) comprised the majority (84%) of the 85 sequences and was found affiliated to the genus *Alcanivorax*. OTU-2 (4 sequences) and OTU-5 (4 sequences) were affiliated to the genus *Marinobacter* and shared 95% sequence identity. OTU-3 (3 sequences) and OTU-4 (3 sequences) were, respectively, affiliated to *Oleibacter* and *Methylophaga*. All other OTUs in the clone library were represented by single sequences and are presented in Supplementary Table 1.

**Table 2:** **SIP-identified sequences in the clone library constructed from ^13^C-enriched DNA^a^**.

**OUT no.**	**Rep. seq.^b^**	**Closest BLASTn match^c^**	**Accessioin no.**	**Clones in library (%)^d^**
1	HEX19	*Alcanivorax jadensis* (98%)	AJ001150	71
2	HEX85	*Marinobacter lipolyticus* (97%)	AY147906	4
3	HEX49	*Oleibacter marinus* (99%)	AB435650	3
4	HEX76	*Methylophaga thiooxydans* (97%)	DQ660915	3
5	HEX17	*Marinobacter flavimaris* (100%)	AY517632	4

a HEX, SIP with [U-^13^C]n-hexadecane.

b Representative sequence for each OTU. Singleton sequences are listed in Supplementary Table 1.

c Results are to the closest type strain; percentage similarity shown in parentheses.

d Total number of sequences in ^13^C-enriched DNA clone library from the n-hexadecane SIP incubation was 85. A 97% cut-off was used to classify sequences to an OTU.

**Figure 3:**
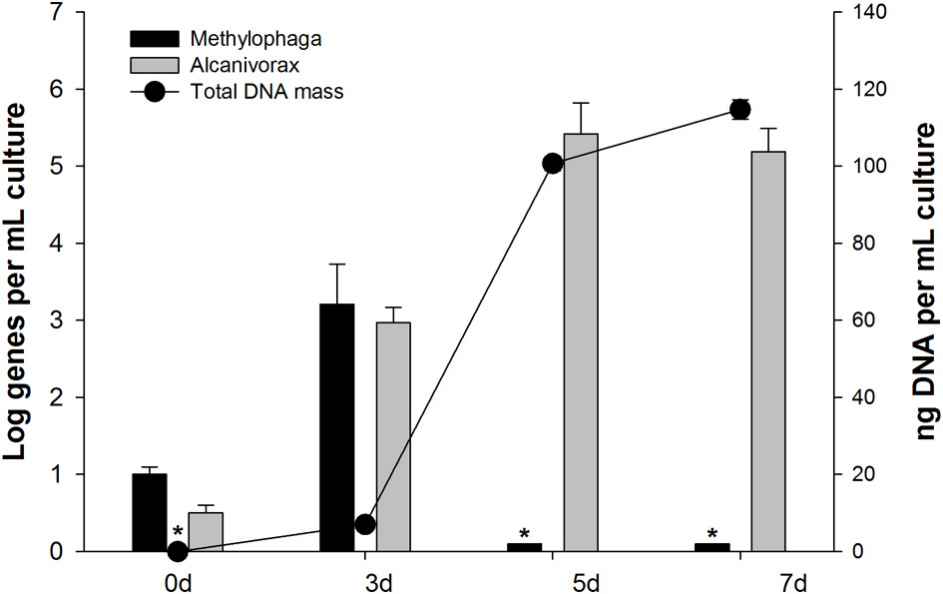
**Abundance of *Alcanivorax* and *Methylophaga* 16S rRNA genes during incubation with unlabeled *n*-hexadecane.** Bars are the mean ± standard deviations of results from triplicate qPCRs measuring the abundance of group-specific 16S rRNA genes. Circles are the mean ± standard deviations of triplicate measurements of the total mass of DNA per sample. Bars or data points with asterisks represent numbers with one or more readings below the quantification limit of the assay and are presented as the largest possible value for that point.

Primers for qPCR were designed targeting the 16S rRNA gene of the most dominant SIP-identified group, *Alcanivorax* OTU-1 (Table 1), in order to quantify this group in the “heavy” DNA from the SIP incubations (Figure 2) and in incubations with unlabeled *n*-hexadecane to confirm its enrichment (Figure 3). As shown in Figure 2, qPCR detection of this group was largely confined to the “heavy” DNA (fractions 7–10), thus confirming its enrichment of the ^13^C label from [^13^C]*n*-hexadecane. By day 5 in the unlabeled incubations (Figure 3), the gene copy number of this group increased by ca. 5 orders of magnitude, coinciding with the disappearance and mineralization of the *n*-hexadecane (Figure 1). This increase in gene copy number also coincided with an increase in the total concentration of DNA as indicator of cell growth. The observed significant increase in the 16S rRNA gene copy number of this organism by day 5 coupled with its growth (total DNA as proxy), the disappearance and mineralization of the *n*-hexadecane, and appearance of respective 16S rRNA genes in only the most heavily ^13^C-enriched DNA fractions (Figure 2) of incubations containing the ^13^C-labeled substrate, strongly supports the enrichment of this *Alcanivorax* on the *n*-hexadecane as a growth substrate.

A similar approach was used to confirm the apparent enrichment of *Methylophaga* OTU-4 in the clone library constructed from ^13^C-enriched DNA (Table 2) and link this organism to the degradation of *n*-hexadecane. As shown in Figure 2, 13% of total *Methylophaga* genes were confined to the “heavy” DNA (fractions 7–10) compared to 56% in the “light” DNA (fractions 16–19), thus indicating partial enrichment of this organism in the ^13^C-enriched DNA. Figure 3 shows that the gene copy number of this group increased by ca. 3 orders of magnitude by day 3, which coincided with ca. 80% disappearance of the *n*-hexadecane, 6% of ^14^C mineralized (as ^14^CO_2_) of initial ^14^C (as ^14^C-labeled *n*-hexadecane) and increase in total DNA concentration (as indicator of growth). At days 5 and 7, the gene copy number of this organism declined to reflect initial numbers (<0.5 Log genes per mL of culture).

### Growth on and degradation of n-hexadecane by *Methylophaga* sp. strain SM14

**Figure 4:**
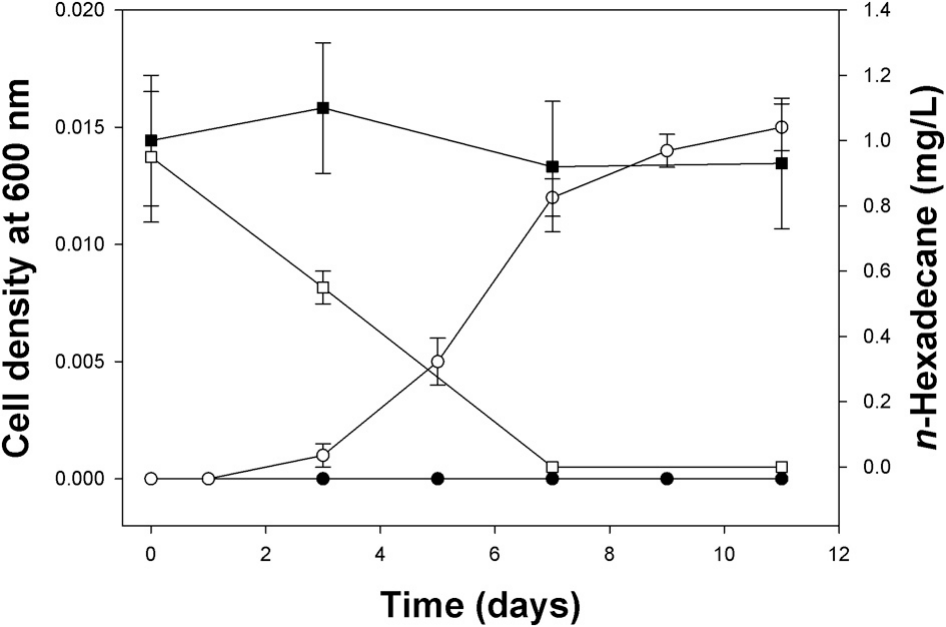
**Growth of *Methylophaga* sp. strain SM14 on *n*-hexadecane (0.002% v/v) as the sole carbon and energy source and disappearance of the *n*-hexadecane as measured by GC.** Each data point is the mean ± standard deviation from triplicate incubations. Open symbols represent live cultures (non-acid treated); filled symbols represent acid-inhibited controls. Circles and squares are cell density and GC measurements, respectively. Some error bars are smaller than the symbol.

Enrichment experiments using the North Carolina field sample as inoculum and with methanol as the sole carbon and energy source yielded two isolates, designated strain SM13 and strain SM14. Both strains were found affiliated to the genus *Methylophaga* based on sequencing of their 16S rRNA gene—they shared 94% sequence identity between them. Strain SM13 proved difficult to maintain in laboratory culture as it eventually ceased to grow upon subsequent subculturing and was therefore no longer used for further experimentation. On the other hand, strain SM14 yielded small (0.05–0.15 mm) off-white colonies after 2 weeks incubation on ONR7a agar amended with Na-pyruvate as the sole carbon and energy source (not shown). The strain also grew well in ONR7a broth amended with Na-pyruvate or methanol. Evidence of the strain's ability to grow on *n*-hexadecane as a sole carbon and energy source is shown in Figure 4. At an initial *n*-hexadecane concentration of 0.002% (v/v), the strain reached a low cell density of ca. 0.015 at 600 nm. However, growth coincided with the disappearance of the *n*-hexadecane, which was indicative that the hydrocarbon was being degraded by the strain and utilized as a carbon source for growth. No growth was measured in uninoculated controls, or in inoculated incubations in the absence of any added *n*-hexadecane. Cultures amended with higher concentrations of *n*-hexadecane yielded higher cell densities (results not shown). Evidence that this was a pure culture of *Methylophaga* strain SM14 was confirmed by 16S rRNA gene sequencing of DNA isolated from cell pellets collected toward the end of these growth experiments.

### Phylogenetic analysis

**Figure 5:**
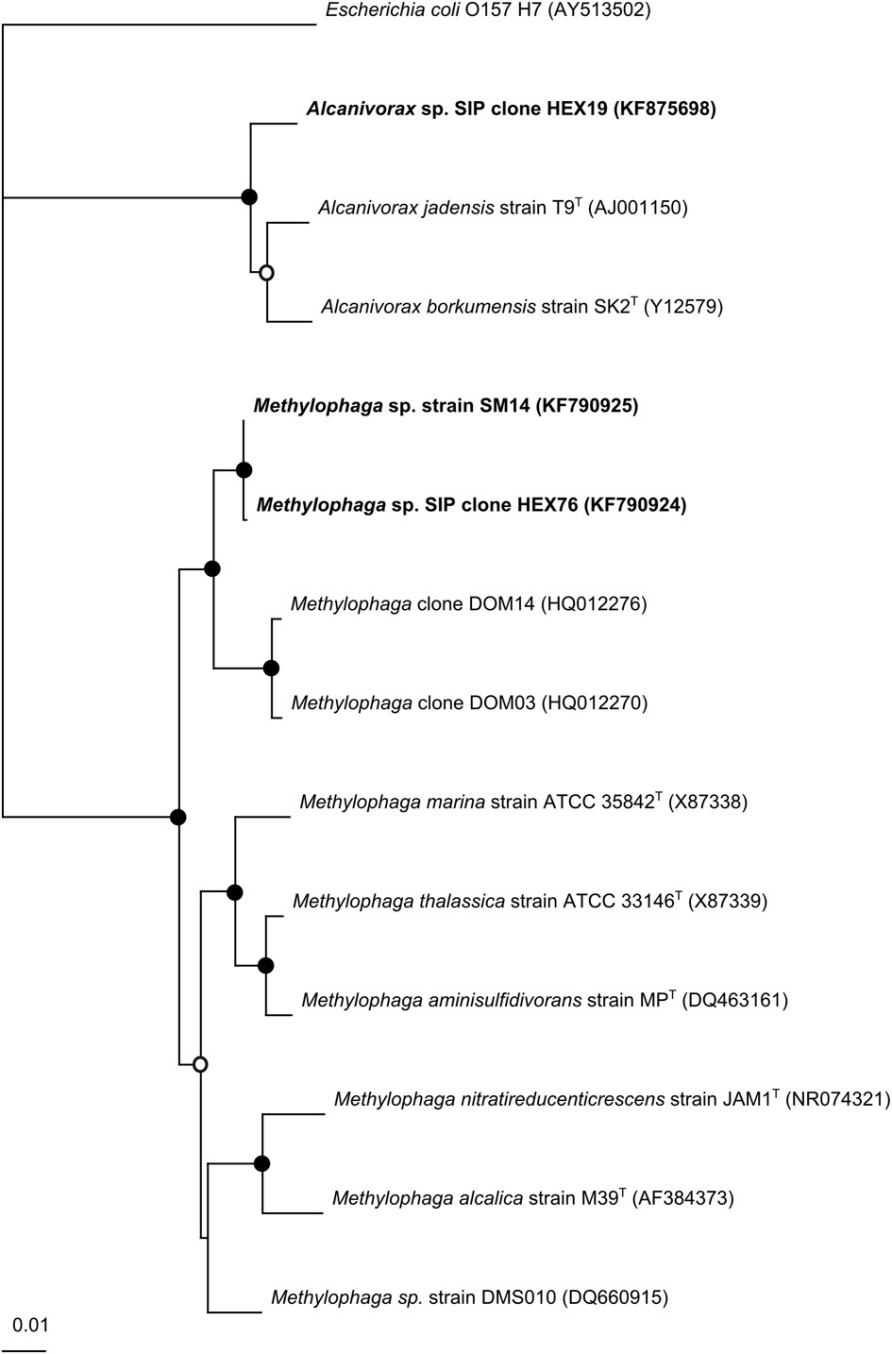
**Phylogenetic tree of SIP clones and the isolated strain from the North Carolina surface seawater sample.** GenBank accession numbers are in parentheses. The tree was constructed using the neighbor-joining algorithm. Nodes with bootstrap support of at least 65% (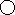) and 90% (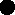) are marked (1000 replications). The scale bar indicates the difference of number of substitutions per site.

The near-complete 16S rRNA gene sequence of the major SIP-identified OTUs and *Methylophaga* strain SM14 were used to construct a phylogenetic tree with related sequences from GenBank (Figure 5). The representative sequence for *Alcanivorax* identified from incubations with ^13^C-hexadecane (HEX19; OTU-1) shared highest 16S rRNA sequence identity (98%) with *Alcanivorax jadensis* T9^T^ (Fernandez-Martinez et al., 2003)—previously (*Fundibacter*) *jadensis* that had been isolated from intertidal sediment collected from the German North Sea coast (Bruns and Berthe-Corti, 1999); second highest sequence identity (97%) was with *Alcanivorax borkumensis* SK2^T^ that was isolated from seawater/sediment samples collected near the Isle of Borkum in the North Sea (Yakimov et al., 1998).

The 16S rRNA sequence of *Methylophaga* strain SM14 shared 99.9% identity with SIP clone HEX76 (*Methylophaga* OTU-4). Highest sequence identity for both SM14 and clone HEX76 was found with two uncultured *Methylophaga* clones, DOM03 and DOM14 (97.5% similarity), identified associated with marine dissolved organic matter (DOM) (McCarren et al., 2010). Highest sequence identity of SM14 and HEX76 to type strains was with *M. thiooxydans* DMS010^T^ (97.1%) isolated from an enrichment culture of the coccolithophorid *Emiliana huxleyi* with dimethylsulfide (Schäfer, 2007; Boden et al., 2010), whereas they shared ≤95% to other *Methylophaga* type strains. To further clarify the phylogenetic identity of strain SM14, tree construction was also performed with the neighbor-joining, maximum parsimony, and maximum likelihood methods. In all cases, the position of strain SM14 always grouped to the genus *Methylophaga*.

## Discussion

SIP has proven useful for linking the phylogenetic identity of microorganisms with a metabolic function. However, careful attention must be employed in its design and execution in order for it to yield interpretable, unambiguous results. One of the main challenges in SIP is obtaining sufficient incorporation of the ^13^C into biomass, which in the case for DNA-SIP, its enrichment into DNA. Whilst the extent of labeling can be increased with longer incubation times, this can lead to the ^13^C becoming distributed among other members of the microbial community—i.e., that are not necessarily directly capable of metabolizing the isotopically-labeled substrate—by cross-feeding on ^13^C-labeled metabolic byproducts, intermediates, or dead cells (Leuders et al., 2004). To avert this, we had set up several ^12^C and ^14^C incubations that ran in parallel to the ^13^C incubations in order to tractably measure for the degradation (by GC-MS) and mineralization (by scintillation counts) of the *n*-hexadecane to help guide our selection of the point at which to terminate the ^13^C incubations (endpoint of experiment) whereby sufficient ^13^C incorporation had been achieved with minimal cross-feeding. The absence of a heavy DNA band in the ^12^C controls (not shown) and the distinct bimodal distribution between ^13^C and ^12^C DNA bands on DGGE confirmed the incorporation of the label from [^13^C]*n*-hexadecane by a subgroup of the total microbial community.

Based on the clone libraries constructed from ^13^C-enriched DNA, the main consumers of the [^13^C]*n*-hexadecane were affiliated with *Alcanivorax*—a group of cosmopolitan bacteria that utilize petroleum oil hydrocarbons almost exclusively as a preferred carbon and energy source. Since this SIP-identified *Alcanivorax*, represented by OTU-1 (clone HEX19), was found with ≥2% 16S rRNA sequence difference to closely related type strains, it is likely to represent a new phylogenetic taxon within this genus. Further confirmation for the enrichment of this *Alcanivorax* OTU and its dominant role in the degradation of the *n*-hexadecane was provided by qPCR which revealed a dramatic increase in the abundance of the 16S rRNA gene copy number for these organisms in the unlabeled incubations (Figure 3). In addition, since growth of these organisms coincided with disappearance of the *n*-hexadecane, and appearance of their 16S rRNA genes in only the most heavily enriched ^13^C-DNA fractions, suggests that their presence in the clone libraries constructed from heavy DNA was unlikely due to cross-feeding. Other possible contributors to the consumption of the [^13^C]*n*-hexadecane during SIP were *Marinobacter* and *Oleibacter*, as several sequences (3–9% of the total clone library) were found affiliated with these genera in the libraries constructed from heavy DNA—members of these genera are commonly found enriched during oil spills in marine waters and can play a major role in the degradation of aliphatic hydrocarbons, such as *n*-hexadecane.

Intriguingly, several sequences affiliated to *Methylophaga* were also identified in the heavy DNA clone libraries (ca. 4% of the total clone library), and since this SIP-identified *Methylophaga* (represented by OTU-4) was found with ≥3% 16S rRNA sequence difference to closely related type strains, it is likely to represent a new phylogenetic taxon within this genus. To our knowledge, this represents the first identification of *Methylophaga* by SIP targeting hydrocarbon degraders using a ^13^C-labeled hydrocarbon. Based on the presence of these organisms in the heavy DNA fractions (Figure 2) and increased abundance of their 16S rRNA gene copy number by day 3 in the unlabeled incubations—which coincided with the disappearance and mineralization of the *n*-hexadecane—these results supported the contribution of the respective newly identified *Methylophaga* (OTU-4) to the degradation of the hydrocarbon. We are unable at the present time to explain why the initial increase in their gene copy number was, by day 5, subsequently followed by a regression in their abundance to below detection limits. We observed similar results during a crude oil enrichment experiment in which pyrosequencing was used to analyse the bacterial community response associated with the marine diatom *Skeletonema costatum* to reveal an initial and distinctive bloom in *Methylophaga* (unpublished results). As the SIP experiment was designed to minimize the possibility of cross-feeding, and the various analyses performed (i.e., DNA quantification, qPCR of target genes, etc) support the enrichment of *Methylophaga* on the *n*-hexadecane as a growth substrate within at least the first 3 days of the experiment, it is highly unlikely that any *Methylophaga* DNA had migrated into the heavy fractions during isopycnic ultracentrifugation unless it was enriched with ^13^C. This is further supported by the fact that no single sequence of the internal standard (unlabeled *E. coli* DNA) was detected in the heavy fractions and clone libraries constructed from the heavy DNA. It is more likely, in fact, that *Methylophaga* sequences were underrepresented in ^13^C-enriched DNA clone libraries because the SIP experiment was terminated (day 7) after the apparent peak in growth of *Methylophaga* occurred (day 3; Figure 3).

In order to further validate whether the SIP-identified *Methylophaga* (OTU-4) were capable of utilizing *n*-hexadecane as a sole carbon and energy source, we isolated a member of this genus, *Methylophaga* strain SM14, that shared 99.9% 16S rRNA gene sequence identity to the representative sequence for this OTU (i.e., SIP clone HEX76) and directly demonstrated its ability to grow on and degrade *n*-hexadecane as a sole carbon and energy source (Figure 4). Collectively, our results present compelling evidence to implicate, for this first time, a novel member of the genus *Methylophaga* in hydrocarbon degradation, hence expanding the substrate spectrum for certain members of this genus beyond solely utilizing C1 carbon sources—with the exception of a few species able to also utilize fructose.

The discovery of hydrocarbon-degrading *Methylophaga* has important implications to assessing their role in the fate of the oil that entered the Gulf of Mexico during the Deepwater Horizon spill. During the active phase of the spill (April 20 to July 15), methylotrophic bacteria (incl. *Methylophaga*) were not detected near the leaky wellhead (Hazen et al., 2010; Valentine et al., 2010; Yang et al., 2014), whereas these organisms were reported to account for at least 5% of the total bacterial community after the spill—i.e., Kessler et al. (2011) reported 5–36% in Sep. 2010; Yang et al. (2014) report 0.23–6% and 2% in Sep. 2010 and Oct. 2010, respectively. However, a recent report by Dubinsky et al. (2013) which analyzed the microbial community composition of plume waters during June and August 2010—a time period not covered in these earlier reports—describes the start of a *Methylophaga* enrichment in plume waters during late June that appeared to be sustained until late August. Furthermore, transcriptional analysis by Rivers et al. (2013) of water column samples collected during the active phase of the spill (May 26 to June 3, 2010) revealed that *Methylophaga* were in a heightened state of metabolic activity within the plume relative to non-plume waters. Kessler et al. (2011) postulated that methylotrophs, such as *Methylophaga*, had contributed to the consumption of the methane released during the spill. However, such organisms are not recognized for carrying out the oxidation, or being capable of growing on, methane as a carbon and energy source. Hence, their potential to have consumed the methane remains contentious in the absence of substantive evidence to support this. Our results from SIP and with strain SM14 herein provide evidence to reassess the possibility that a subset of the *Methylophaga* community in the Gulf of Mexico, specifically those with hydrocarbon-degrading potential, may have contributed to the degradation of some components of the oil (e.g., saturated hydrocarbons). Dubinsky et al. (2013) measured higher-than-background levels of BTEX, cyclo-alkanes and *n*-alkanes in plume waters during the *Methylophaga*-enrichment phase in late June that, hence, might have acted as potential carbon and energy sources for these organisms. We hypothesize that the apparent enrichment of these organisms, albeit ephemerally, during the latter phase of the spill may have been in part associated with the potential for some members of this genus to degrade hydrocarbons.

## Author contributions

Sara Mishamandani, Tony Gutierrez, and Michael D. Aitken contributed to the design of the work and its interpretation. Sara Mishamandani and Tony Gutierrez produced all of the data and, together with Michael D. Aitken, drafted and critically revised the manuscript and approved the final version for publication.

### Conflict of interest statement

The authors declare that the research was conducted in the absence of any commercial or financial relationships that could be construed as a potential conflict of interest.

## Supplementary material

Supplementary Table 1 **Singleton 16S rRNA gene sequences recovered from the heavy DNA clone library^a^**.
