# Supplementary material for: DNA-based stable isotope probing coupled with cultivation methods implicates Methylophaga in hydrocarbon degradation
Source: Front Microbiol. 2014 Feb 27;5:76. doi: 10.3389/fmicb.2014.00076 (PMC3936186; doi:10.3389/fmicb.2014.00076)
Supplement: Supplementary Table 1 — Singleton 16S rRNA gene sequences recovered from the heavy DNA clone librarya. [file DataSheet1.PDF]

Supplementary table 1. Singleton 16S rRNA gene sequences recovered from the heavy DNA clone library.[a]

OTU No.	Clone name	Closest BLASTn match[b]	Accession No.
6	HEX11	*Alteromonas genovensis* (96%)	AM887686
7	HEX02	*Sandarakinotalea sediminis* (99%)	AB206955
8	HEX67	*Blastomonas ursincola* (96%)	AB024289
9	HEX52	*Silicibacter pomeroyi* (100%)	AF434674
10	HEX03	*Fundibacter jadensis* (95%)	AJ001150
11	HEX40	*Phaeobacter gallaeciensis* (98%)	KC176239

[a] HEX, SIP with [U-^{13}C]*n*-hexadecane.
[b] Results are to the closest type strain; percentage similarity shown in parentheses.
